# Adsorption and Aggregation Properties of Some Polysorbates at Different Temperatures

**DOI:** 10.1007/s10953-018-0823-z

**Published:** 2018-10-26

**Authors:** Katarzyna Szymczyk, Anna Zdziennicka, Bronisław Jańczuk

**Affiliations:** Department of Interfacial Phenomena, Faculty of Chemistry, Maria Curie-Skłodowska University, Maria Curie-Skłodowska Sq. 3, 20-031 Lublin, Poland

**Keywords:** Adsorption, Micellization, Polysorbates, Surface tension, Thermodynamic functions

## Abstract

Measurements of the surface tension of aqueous solutions of polysorbates (Tween 20, Tween 60 and Tween 80) at 293, 303 and 313 K were made. On the basis of the obtained results the Gibbs surface excess concentration of the Tweens at the water–air interface and critical micelle concentrations were determined. Knowing the Gibbs surface excess concentration and taking into account the difference between the limiting area occupied by water and Tween molecules at the water–air interface, the fraction occupied by Tween molecules was established. The limiting area occupied by the Tween molecule was calculated by applying the Joos equation. The area determined in such a way was confirmed by the calculations of cross section of Tween molecules based on the bond lengths and the angles between them as well as the average distance between the molecules, taking into account their different conformations. This area was used for calculation of the standard Gibbs energy of adsorption using the Langmuir equation. The standard Gibbs energy of Tweens adsorption at the water–air interface was also calculated from the hydrophobic part of Tween molecule–water interface tension and that of hydrophobic part. Using the determined values of standard Gibbs energy of adsorption at different temperatures, the standard enthalpy and entropy values were deduced. The standard thermodynamic functions of micellization were also determined and compared to the Gibbs energy of Tween molecules interactions through the water phase.

**Electronic supplementary material:**

The online version of this article (10.1007/s10953-018-0823-z) contains supplementary material, which is available to authorized users.

## Introduction

Polysorbates, among others are applied in the food, cosmetic and pharmaceutical industries because of their nontoxicity, availability, emulsification, solubilization and other advantages [1–7]. Of the polysorbates, Tween 20 (T20), Tween 60 (T60) and Tween 80 (T80) play the most important role in practical applications. T20 and T60 are capable of enhancing drug transport across biological membranes [5]. Also, T80 is widely used in drug dosage forms to control wetting, stability and solubilization of hydrophobic drugs [3, 4, 6, 7]. However, their wide practical applications are not completely explained, taking into account their adsorption and aggregation properties in the aqueous media. Moreover, in the literature it is possible to find some discrepancies in these properties [8–17].

It should be remembered that the structures of T20, T60 and T80 molecules are complicated and they can be treated as short chain polymeric surfactants. The molecules of these Tweens are composed of four polyoxyethylene chains, a hydrocarbon chain connected with polyoxyethylene one and a heterocyclic ring. These four polyoxyethylene chains are joined to the heterocyclic ring. Depending on the conformation of polyoxyethylene chains, the adsorption and aggregation properties of Tweens are probably different. The difference depends on the environmental conditions. Probably for these reasons, investigators have obtained different values of the Gibbs surface excess concentration in the monolayer at the water–air interface and different values of critical micelle concentrations (CMC) [8–17]. Indeed, the CMC values of Tweens strongly depend on the method of their determination. Thus, literature reports the CMC data obtained from the surface tension isotherms [8, 10–12, 14, 16, 17], generally determined from the values of aqueous Tween solution surface tensions measured by the ring method [8, 10, 11]. It is known that in the case of aqueous surfactant solutions, each method can give a somewhat different result [8–19]. This occurs particularly in the case of surfactants of large molecular weight. It should be also mentioned that it is difficult to find thermodynamics functions describing the adsorption and micellization processes of Tweens in the literature. On the other hand, there are a only few data dealing with the relationship between the surface tension of Tweens and their adsorption and aggregation properties [8–17]. As is commonly known, the surface tension of surfactants depends on the orientation of their molecules towards the air phase [20, 21]. If they are oriented with the hydrophobic part towards the air, then the surfactant surface tension practically results from only the Lifshitz–van der Waals intermolecular interactions. However, if the surfactant molecules are oriented with the hydrophilic part towards the air, then the surfactants surface tension results not only from the Lifshitz–van der Waals intermolecular interactions but also from the Lewis acid–base interactions. The purpose of our studies was to determine the adsorption of Tweens at the water–air interface and their CMCs based on the surface tension of the hydrophobic (tail) and hydrophilic (head) parts of the Tweens. For this purpose, the surface tensions of the aqueous solutions of T20, T60 and T80 at 293, 303 and 313 K were measured. On the basis of the obtained values of surface tension, the thermodynamic analysis of the adsorption and micellization processes of Tweens was performed.

## Experimental

### Materials

**Fig. 1:**
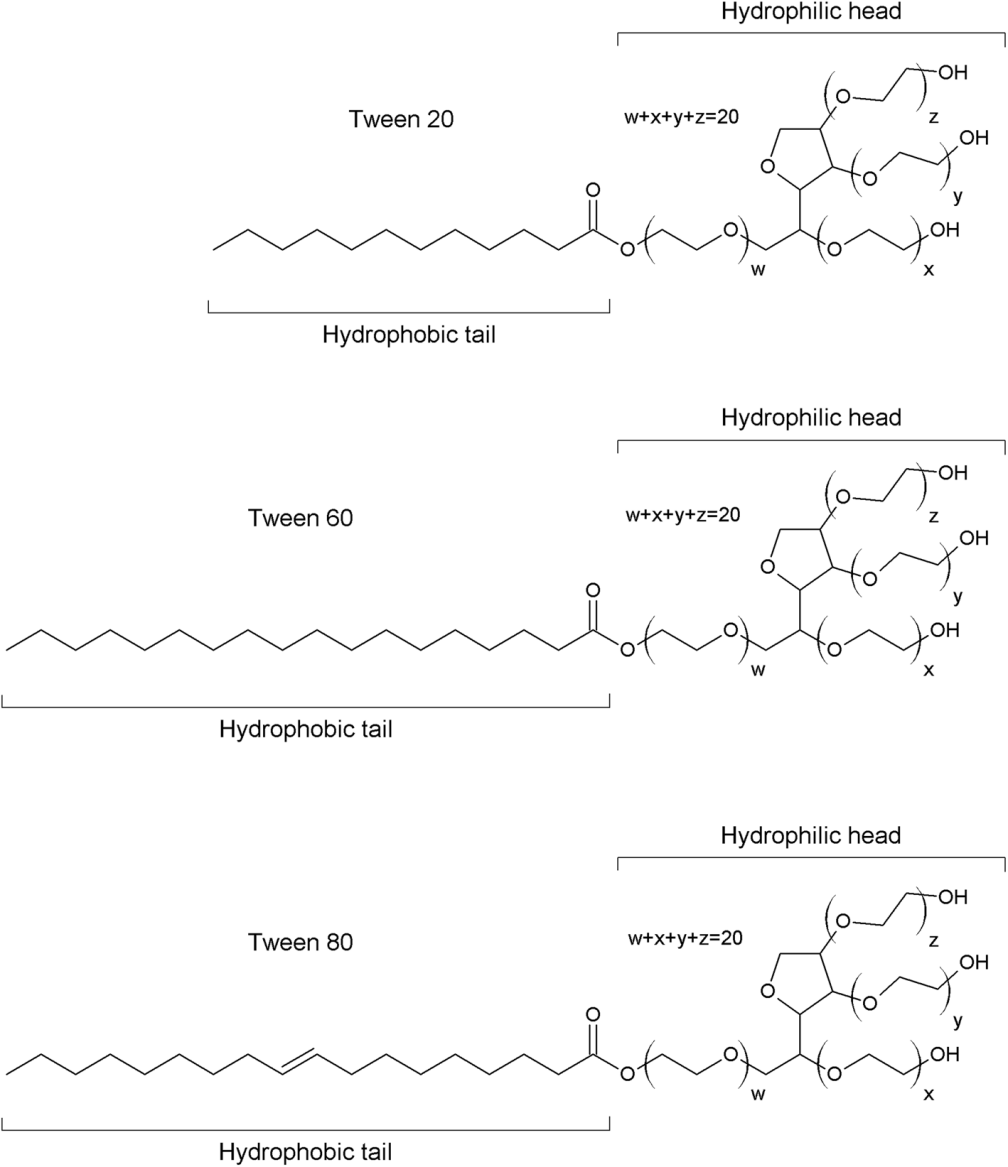
The structures of Tween 20 (T20), Tween 60 (T60) and Tween 80 (T80)

In our studies polyethylene glycol sorbitan monolaurate, Tween^**®**^ 20 (T20) (Sigma–Aldrich; CAS: 9005-65-5; lauric acid, ≥ 40%, balance primarily myristic, palmitic, and stearic acids), polyethylene glycol sorbitan monostearate, Tween^**®**^ 60 (T60) (Sigma–Aldrich; CAS: 9005-67-8; stearic acid, 40–60%, total stearic and palmitic acid, ≥ 90%) and polyethylene glycol sorbitan monooleate, Tween^**®**^ 80 (T80) (Sigma–Aldrich: CAS: 9005-65-6; oleic acid, ≥ 58.0%, balance primarily linoleic, palmitic, and stearic acids) were used without purification. The structure of Tweens is presented in Fig. 1.

The water used for solution preparation and surface tension measurements was doubly distilled and deionized (Destamat Bi18E) and its resistance was equal to 18.2 MΩ·cm. The purity of water was also checked by surface tension measurements.

All the aqueous solutions of Tweens at a given concentration lower than 1 × 10^−2^ mol·dm^−3^ were prepared from the stock solution (1 × 10^−2^ mol·dm^−3^). Then from this solution the others in the concentration range from 1 × 10^−3^ to 1 × 10^−2^ mol·dm^−3^ were prepared. Next, from the solution at the concentration equal to 1 × 10^−3^ mol·dm^−3^, the solutions in the concentration range from 1 × 10^−4^ to 1 × 10^−3^ mol·dm^−3^ were prepared and so on. The stock solution was prepared by weight using an analytical balance (model XA105, Mettler–Toledo) with a precision of ± 0.01 mg. The standard uncertainties (u(*C*)) changed from 9.1 × 10^−12^ to 9.1 × 10^−7^ mol·dm^−3^ for Tweens in the range of the concentrations studied.

### Surface Tension Measurements

The equilibrium surface tensions $\left( {\gamma_{\text{L}} } \right)$ of the aqueous solutions of T20, T60 and T80 were first measured using the Krüss K100 tensiometer by the ring method. The tensiometer was calibrated especially by taking into account the local earth’s gravitational acceleration and the procedure of Huh and Mason [22] and was tested against water ($\gamma_{\text{L}} = 7 2. 8 {\text{ mN}}\cdot{\text{m}}^{ - 1}$) and methanol ($\gamma_{\text{L}} = 2 2. 5 {\text{ mN}}\cdot{\text{m}}^{ - 1}$). The ring was cleaned with distilled water and heated to red heat with a Bunsen burner before each measurement. In all cases 10 or more successive measurements were performed. It appeared that the values of the surface tensions of aqueous solutions of Tweens at a given concentration, measured by the tensiometric method, increased with increasing repetitions of the measurement, for the same sample of the solution. Therefore, it was difficult to establish the real value of surface tensions of the aqueous solutions of Tweens, particularly at its low concentration. Thus, the equilibrium surface tensions of the solutions were determined by the DSA30 measuring system (Krüss) using the weight–volume method at 293, 303 and 313 K. Before the measurements of surface tension, the solution of Tween at a given concentration was placed in the internal thermostated chamber for 2 h and then inserted into the measuring chamber. The measurement temperature was controlled by a jacketed vessel joined to a thermostatic water bath with an accuracy of ± 0.1 K. For measurements of the surface tensions of aqueous solutions of Tweens by the weight–volume method, different capillaries were used depending on the concentrations of the Tweens. At each concentration, the cross section radius of the capillary was such that the drop detached from the total surface of its top. Additionally, at a low concentration of Tweens the value of the capillary radius was checked by measurements of the water surface tension at a given temperature and at high concentrations using methanol. On the basis of the image, the moment of drop detachment was controlled. For each concentration of the aqueous solutions of Tweens more than 10 successive measurements were made. The root-mean-square deviation $\left( {\left( {\frac{1}{n - 1}\sum\limits_{k = 1}^{n} {\left( {X_{i,k} - \overline{X}_{i} } \right)^{2} } } \right)^{1/2} } \right)$ (where *n* is the series of measurements at a given surfactant concentration, $X_{i,k}$ is the measured value and $\overline{X}_{i}$ is the average value of the surface tension at a given surfactant concentration) of our surface tension data depending on the surfactant concentration and was in the range from ± 0.1 to ± 0.25 mN·m^−1^ and the standard uncertainty (standard deviation of the mean, $\left( {\frac{1}{n(n - 1)}\sum\limits_{k = 1}^{n} {\left( {X_{i,k} - \overline{X}_{i} } \right)^{2} } } \right)^{1/2}$), was in the range from ± 0.025 mN·m^−1^ (calculated for 16 surface tension values for each surfactant concentration in the range of its low concentration) to ± 0.079 mN·m^−1^ (calculated for 10 surface tension values for each surfactant concentration in the range of its high concentration), respectively. The accuracy of the DSA30 measuring system, resulting from instrument accuracy and the temperature measurements, equals 0.03 mN·m^−1^ and considering the number of performed measurements, the total uncertainty was in the range from 0.1 to 0.17 mN·m^−1^.

## Results and Discussion

### Adsorption of Tweens at the Water–Air Interface

**Fig. 2:**
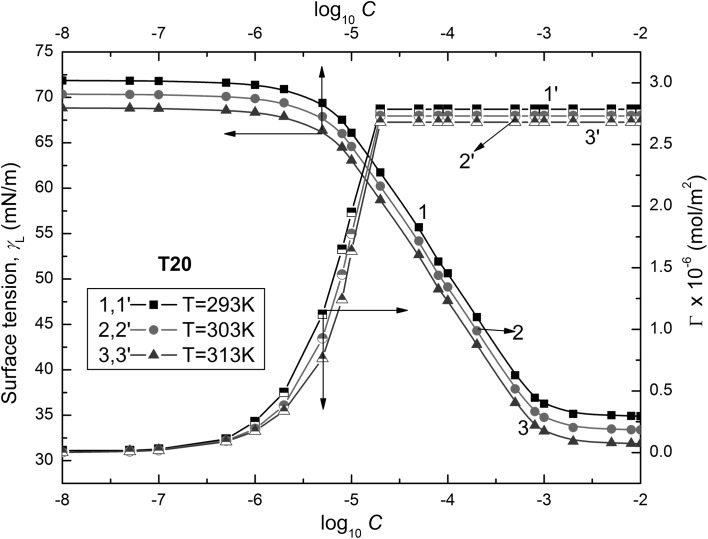
Plots of the surface tensions of the aqueous solutions of T20 ($\gamma_{\text{L}}$) (curves 1–3) and the Gibbs surface excess concentration ($\varGamma_{{}}$) calculated from Eq. 1 (curves 1′–3′) against the logarithm of the Tween concentration (log_10_ *C*). Curves 1 and 1′ correspond to 293 K, curves 2 and 2′ to 303 K and curves 3 and 3′ to 313 K

**Fig. 3:**
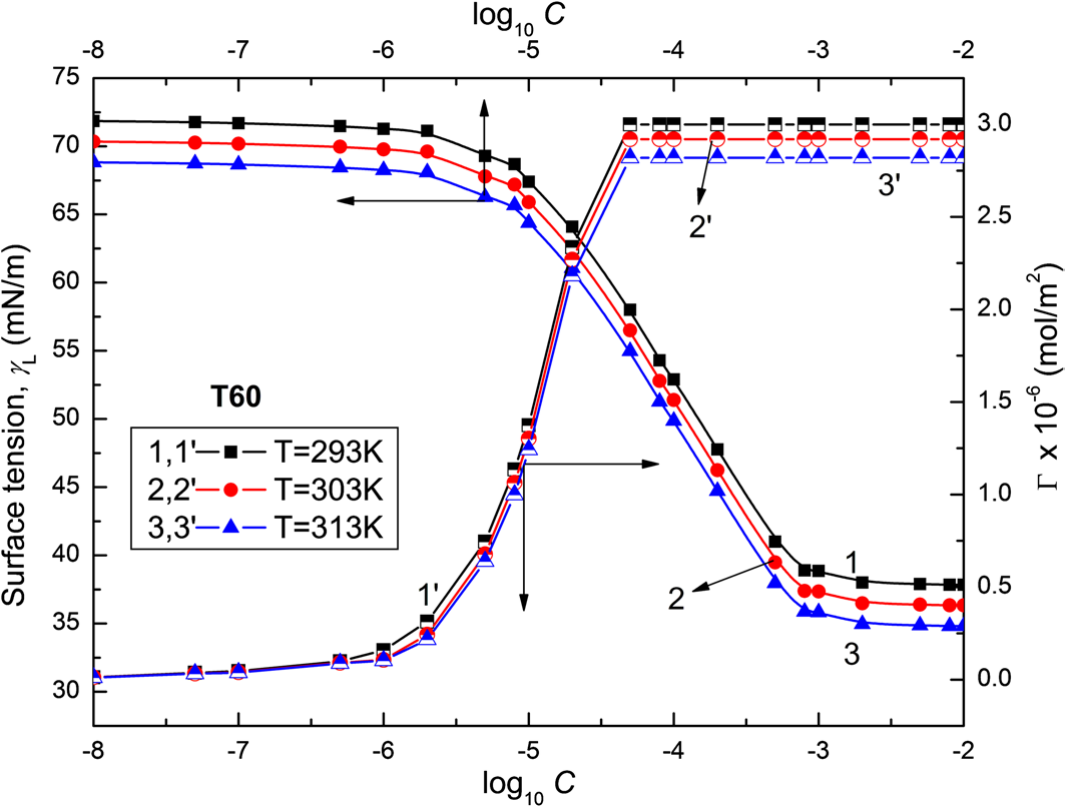
Plots of the surface tensions of the aqueous solutions of T60 ($\gamma_{\text{L}}$) (curves 1–3) and the Gibbs surface excess concentration ($\varGamma_{{}}$) calculated from Eq. 1 (curves 1′–3′) against the logarithm of Tween concentration (log_10_ *C*). Curves 1 and 1′ correspond to 293 K, curves 2 and 2′ to 303 K, and curves 3 and 3′ to 313 K

**Fig. 4:**
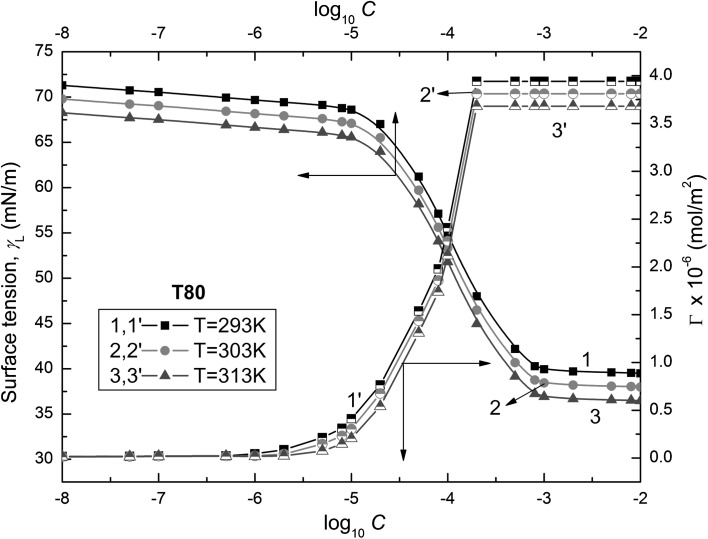
Plots of the surface tensions of the aqueous solutions of T80 ($\gamma_{\text{L}}$) (curves 1–3) and the Gibbs surface excess concentrations ($\varGamma_{{}}$) calculated from Eq. 1 (curves 1′–3′) against the logarithm of Tween concentration (log_10_ *C*). Curves 1 and 1′ correspond to 293 K, curves 2 and 2′ to 303 K and curves 3 and 3′ to 313 K

Practical applications of surfactants result, among others, from their adsorption at the water–air interface. However, in the literature there are only a few direct methods for determination of the surfactant amount at this interface [23]. Therefore, in most cases the surfactant adsorption at the water–air interface is determined using the Gibbs isotherm equation, which is based on the surface tension measurements of aqueous solutions of surfactants. The isotherms of surface tension ($\gamma_{\text{L}}$) of Tween aqueous solutions (Figs. 2, 3, and 4) are similar to those of classical nonionic surfactants [24].

These isotherms show minimal values of surface tension at the CMC, indicating that the Tweens used by us are practically impurity free. It should also be mentioned that there is a linear dependence between the surface tensions of the solutions and the logarithm of surfactant concentration in the range corresponding to the saturated monolayer at the water–air interface. Assuming that the Tween activity coefficients in the studied concentration range are close to unity, it is possible to calculate the Gibbs surface excess concentration from the following equation [18]:

1 $$\varGamma = \frac{{C_{i} }}{RT}\left( {\frac{{\partial \gamma_{\text{L}} }}{\partial C}} \right)_{T} = - \frac{1}{RT}\left( {\frac{{\partial \gamma_{\text{L}} }}{\partial \ln C}} \right)_{T} = \frac{1}{2.303RT}\left( {\frac{{\partial \gamma_{\text{L}} }}{{\partial \log_{10} C}}} \right)_{T}$$

where $\varGamma_{{}}$ is the Gibbs surface excess concentration, *C* is the concentration of surfactant, *R* is the gas constant and *T* is the absolute temperature.

To solve Eq. 1 for $\varGamma_{{}}$, the function describing the dependence between $\gamma_{\text{L}}$ and log_10_ *C* should be known. It proves that in the Tween range concentration corresponding to the unsaturated monolayer at the water–air interface, the change of $\gamma_{\text{L}}$ of the solution, as a function of concentration, can be expressed by a first or second order exponential function. Therefore, it is easy to calculate $\frac{{{\text{d}}\gamma_{\text{L}} }}{{{\text{d}}C}}$. However, in the range of Tween concentration corresponding to the saturated monolayer at the water–air interface, Eq. 1 can be solved by determination of $\frac{{{\text{d}}\gamma_{\text{L}} }}{{{\text{dlog}}_{10} C}}$ because there is a linear dependence between $\gamma_{\text{L}}$ and log_10_ *C* in this range of *C*. The calculated values of $\varGamma_{{}}$ depend on the kind of Tween (Figs. 2, 3, and 4). The maximal values of $\varGamma_{{}}$($\varGamma_{\hbox{max} }^{{}}$) increase from T20 to T80 (Table 1). For each Tween, these values decrease with temperature increase. It is difficult to compare our maximal values of $\varGamma_{{}}$ for Tweens to those in the literature because in most cases they were determined from the surface tension measurements by the ring or Wilhelmy plate methods [8, 10, 12, 16, 17]. However, only in the case of Tween 20 are these values comparable but they are considerably higher for T60 and T80. Thus, the question arises whether the maximal values of $\varGamma_{{}}$ obtained by us are possible for such surfactants as Tweens having branched hydrophilic parts. The reply can be obtained from the calculations of the limiting area occupied by the Tween molecule at the water–air interface. It is possible to establish this area, among others, by applying the Joos equation and taking into consideration the structure of Tween molecules based on the bond length and angle between them as well as the average distance between the molecules, assuming their different conformations.

**Table 1:** The values of maximal ($\varGamma_{\hbox{max} }^{{}}$) and limiting ($\varGamma_{{}}^{\infty }$) Gibbs surface excess concentrations of Tweens calculated from Eqs. 1 and 2, respectively, and the corresponding values of area (*A*) and limiting area ($A_{0}$) calculated from Eq. 3 and the fraction of the surface occupied by Tweens at the water–air interface calculated from the ratio $\varGamma /\varGamma^{\infty }$($X_{1}$) and from Eq. 5 ($X_{1}^{s}$), respectively

	*T* = 293 K	*T* = 303 K	*T* = 313 K
T20			
$\varGamma_{\hbox{max} }^{{}}$ [× 10^6^ mol·m^−2^]	2.79	2.73	2.68
*A* [Å^2^]	59.51	60.82	61.95
$\varGamma_{{}}^{\infty }$ [× 10^6^ mol·m^−2^]	3.63	3.56	3.46
$A_{0}$[Å^2^]	45.74	46.64	47.99
$X_{1}$	0.7672	0.7669	0.7746
$X_{1}^{\text{s}}$	0.7672	0.7671	0.7746
T60			
$\varGamma_{\hbox{max} }^{{}}$ [× 10^6^ mol·m^−2^]	3.00	2.92	2.82
*A* [Å^2^]	55.34	56.86	58.88
$\varGamma_{{}}^{\infty }$ [× 10^6^ mol·m^−2^]	3.61	3.49	3.38
$A_{0}$[Å^2^]	45.99	47.57	49.12
$X_{1}$	0.8310	0.8367	0.8343
$X_{1}^{\text{s}}$	0.8310	0.8366	0.8343
T80			
$\varGamma_{\hbox{max} }^{{}}$ [× 10^6^ mol·m^−2^]	3.94	3.81	3.68
*A* [Å^2^]	42.14	43.58	45.12
$\varGamma_{{}}^{\infty }$ [× 10^6^ mol·m^−2^]	4.04	3.9	3.77
$A_{0}$[Å^2^]	41.10	42.57	44.04
$X_{1}$	0.9752	0.9769	0.97612
$X_{1}^{\text{s}}$	0.9753	0.9769	0.9761

The Joos equation can be written in the form [25]:

2 $$\exp \left( {\frac{ - \varPi }{{RT\varGamma_{\text{W}}^{\infty } }}} \right) + \exp \left( {\frac{ - \varPi }{{RT\varGamma_{\text{S}}^{\infty } }}} \right)\frac{C}{{a_{\text{S}} }} = 1$$

where $\varPi = \gamma_{\text{W}} - \gamma_{\text{L}}$ ($\gamma_{\text{W}}$ is the water surface tension), $\varGamma_{\text{W}}^{\infty }$ and $\varGamma_{\text{S}}^{\infty }$ are the possible limiting Gibbs surface excess concentrations of water and surfactant, respectively, and $a_{\text{S}} = \exp \left( {\frac{{\mu_{\text{S}}^{{0,{\text{S}}}} - \mu_{\text{S}}^{{0,{\text{B}}}} }}{RT}} \right)\omega$ (*ω* is the number of water moles in 1 dm^3^, $\mu_{\text{S}}^{{0,{\text{S}}}}$ and $\mu_{\text{S}}^{{0,{\text{B}}}}$ are the standard potentials of surfactant in the surface layer and bulk phase).

Knowing $\varGamma_{{}}^{\infty }$ it is possible to calculate the limiting area ($A_{0}$) from the following expression:

3 $$A_{0} = \frac{1}{{\varGamma^{\infty } N}}$$

where *N* is the Avogadro number.

The values of $\varGamma_{{}}^{\infty }$ calculated from Eq. 2 for all Tweens at each temperature are higher than $\varGamma_{\hbox{max} }^{{}}$.

**Fig. 5:**
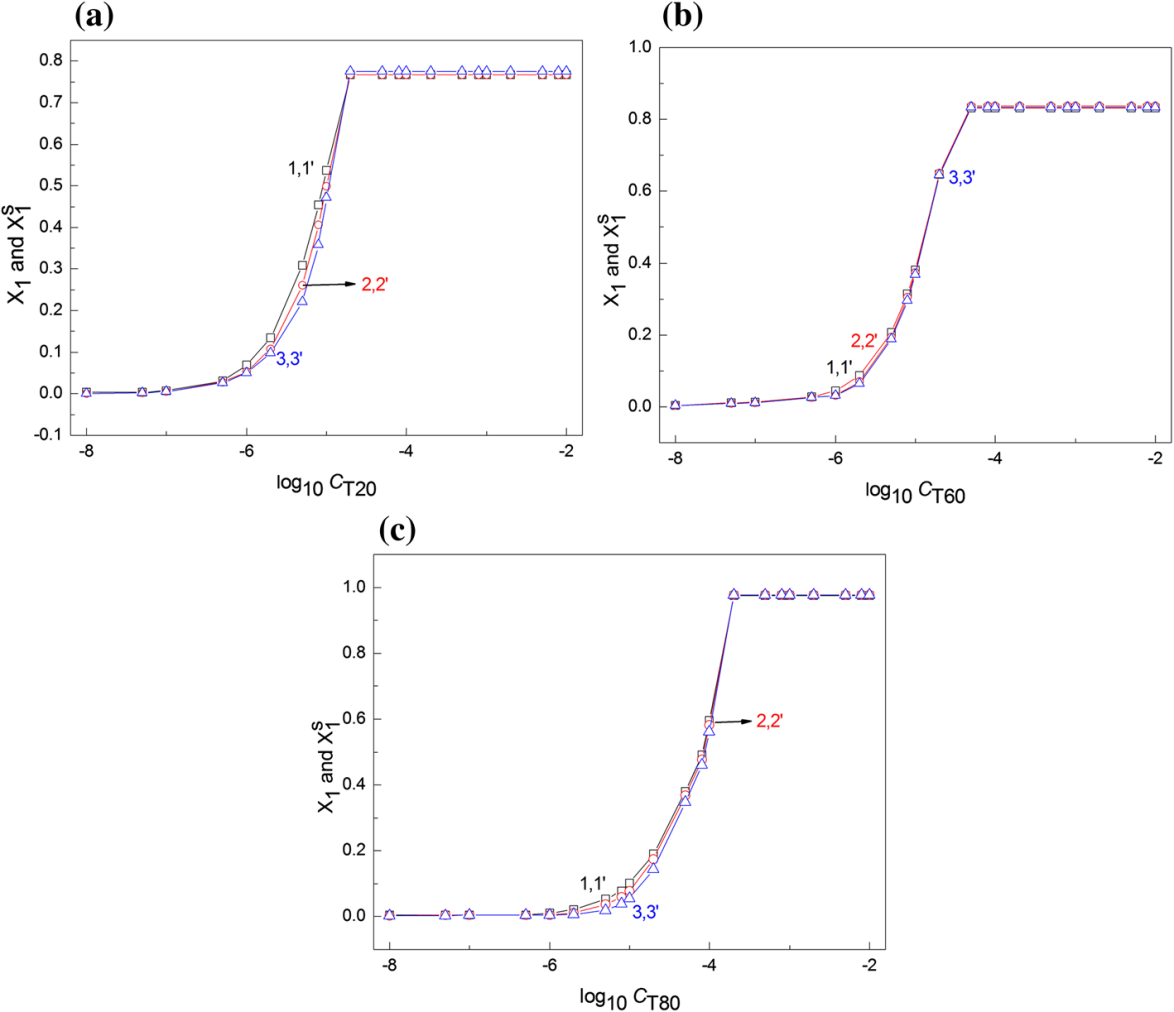
A plot of the mole fraction occupied by T20 (**a**), T60 (**b**) and T80 (**c**) at the water–air interface calculated from the ratio $\varGamma /\varGamma^{\infty }$ ($X_{1}$) (curves 1–3) and calculated from Eq. 5 ($X_{1}^{\text{S}}$) (curves 1′–3′) against the logarithm of their concentration (log_10_ *C*). Curves 1 and 1′ correspond to 293 K, curves 2 and 2′ to 303 K and curves 3 and 3′ to 313 K

Unfortunately, on the basis of $\varGamma_{{}}^{\infty }$ calculated from Eq. 2, it is impossible to state whether its value is comparable to $\varGamma_{{}}$ resulting from the size of the given Tween molecule. To explain this problem the volume of the Tweens molecule was determined from the bond lengths, the angles between them and the average distance between surfactant molecules and water molecules. The volumes of T20, T60 and T80 molecules calculated in this way are in the ranges 1895.49–2019.48, 2031.16–2180.72 and 2028.05–2117.97 Å^3^, respectively, depending on the conformations of the Tween molecules. It is interesting that the volume of the Tween molecules calculated in this way are close to those calculated based on their molecular weight and density. It follows from the calculations that the cross sectional area should be in the range 34.60–67.64 Å^2^, in agreement with all the *A*_0_ values determined from the Joos equation This indicates that the $\varGamma_{\hbox{max} }^{{}}$ values determined from Eq. 1 are reasonable. Knowing the values of $\varGamma_{{}}^{\infty }$ it is possible to determine the fraction of surface occupied by the Tween molecules at the water–air interface ($X_{1}$). It is equal to $\varGamma /\varGamma^{\infty }$. The values of $X_{1}$ calculated in this way are presented in Fig. 5. It can be seen that there are weak repulsive interactions and/or weak attractive interactions between the heads of Tween molecules in the saturated monolayer at the water–air interface as the maximal values of $X_{1}$ are close to 0.77, 0.83 and 0.98 for T20, T60 and T80, respectively, and practically do not depend on the temperature in the studied range. The literature reports the calculation of fraction of interface covered by surfactant $\left( {X_{i}^{\text{S}} } \right)$ (from the following equation [26]:

4 $$X_{i}^{\text{S}} = \frac{{\varGamma_{1} }}{{\varGamma_{\text{W}} + \varGamma_{1} }}$$

where the index W refers to water.

The values of $X_{i}^{\text{S}}$ calculated from Eq. 4, not presented here, are significantly different from those obtained from $\varGamma /\varGamma^{\infty }$. Thus the question arises why there are great differences between the values determined in the two ways. Equation 4 is accurate only for the case where the limiting area of the water and surfactant molecules are the same. In the other cases, as established earlier by us [26], for calculation of $X_{1}$ the ratio of the limitng area occupied by the surfactant molecule to water should be taken into account and then Eq. 4 should be modified to the form:

5 $$X_{1}^{\text{S}} = \frac{{\varGamma_{1} }}{{k\varGamma_{\text{W}} + \varGamma_{1} }}$$

where $$\frac{{\varGamma_{ 1}^{\infty } }}{{\varGamma_{\text{W}}^{\infty } }} = \frac{1}{k}$$.

Indeed, to calculate $X_{i}^{\text{S}}$ from Eq. 5 the values of $\varGamma_{\text{W}}$ should be known. $\varGamma_{\text{W}}$ can be calculated from the following expression [27]:

6 $$\varGamma_{\text{W}} NA_{\text{W}}^{0} + \varGamma_{1} NA_{1} = 1$$

where $A_{\text{W}}^{0}$ and $A_{1}$ are the minimal surface areas per molecule of water and surfactant, respectively.

Introducing the values of $\varGamma_{\text{W}}$ calculated from Eq. 6 and $\varGamma$ from Eq. 1 as well as *k* to Eq. 5, $X_{1}^{\text{S}}$ was determined and is presented in Fig. 5. It appears that the $X_{1}^{\text{S}}$ values obtained from Eq. 5 are almost the same as those obtained on the basis of $\varGamma /\varGamma^{\infty }$.

### Thermodynamic Functions of Tweens Adsorption at the Water–Air Interface

The standard thermodynamic functions such as Gibbs energy, enthalpy and entropy indicate whether the adsorption process is spontaneous and what happens during this process. It is commonly known that under exothermic and isobaric conditions the standard Gibbs energy of adsorption, $\Delta G_{\text{ads}}^{\text{o}},$ can be expressed as [18, 28]:

7 $$\Delta G_{\text{ads}}^{\text{o}} = \Delta H_{\text{ads}}^{\text{o}} - T\Delta S_{\text{ads}}^{\text{o}}$$

where $\Delta H_{\text{ads}}^{\text{o}}$ is the standard enthalpy and $\Delta S_{\text{ads}}^{\text{o}}$ is the standard entropy of adsorption.

Knowing the values of $\Delta G_{\text{ads}}^{\text{o}}$ at different temperatures, it is possible to calculate $\Delta H_{\text{ads}}^{\text{o}}$ and $\Delta S_{\text{ads}}^{\text{o}}$. If it is assumed that in a range of temperature, $\Delta H_{\text{ads}}^{\text{o}}$ is constant then:

8 $$\frac{{{\text{d}}\left( {\Delta G_{\text{ads}}^{\text{o}} } \right)}}{{{\text{d}}T}} = - \Delta S_{\text{ads}}^{\text{o}}$$

On the other hand, if $\Delta S_{\text{ads}}^{\text{o}}$ is constant, then:

9 $$T^{2} \frac{{{\text{d}}\left( {\frac{{\Delta G_{\text{ads}}^{\text{o}} }}{T}} \right)}}{{{\text{d}}T}} = - \Delta H_{\text{ads}}^{\text{o}}$$

In the literature there are many methods for the calculation of $\Delta G_{\text{ads}}^{\text{o}}$. However, the Langmuir [18, 28] equation modified by de Boer [29] is most frequently applied. This equation has the form:

10 $$\frac{{A_{0} }}{{A - A_{0} }}\exp \left( {\frac{{A_{0} }}{{A - A_{0} }}} \right) = \frac{C}{\omega }\exp \left( {\frac{{ - \Delta G_{\text{ads}}^{\text{o}} }}{RT}} \right)$$

$\Delta G_{\text{ads}}^{\text{o}}$ can be also calculated directly from the linear form of the Langmuir equation [18]:

11 $$\frac{{C{}_{{}}}}{\varGamma } = \frac{C}{{\varGamma_{\hbox{max} }^{{}} }} + \frac{a}{{\varGamma_{\hbox{max} }^{{}} }}$$

knowing that:

12 $$a = \omega \exp \left( {\frac{{\Delta G_{\text{ads}}^{\text{o}} }}{RT}} \right)$$

In the literature it is also possible to find the calculation of $\Delta G_{\text{ads}}^{\text{o}}$ from the Rosen equation which has the form [18]:

13 $$\Delta G_{\text{ads}}^{\text{o}} = RT\ln \left( {\frac{{C_{\text{CMC}} }}{\omega }} \right) - \frac{{\pi_{e} }}{{\varGamma_{ \hbox{max} } }}$$

where $\pi_{e}$ is the difference between the water and surfactant solution surface tension at the CMC.

**Fig. 6:**
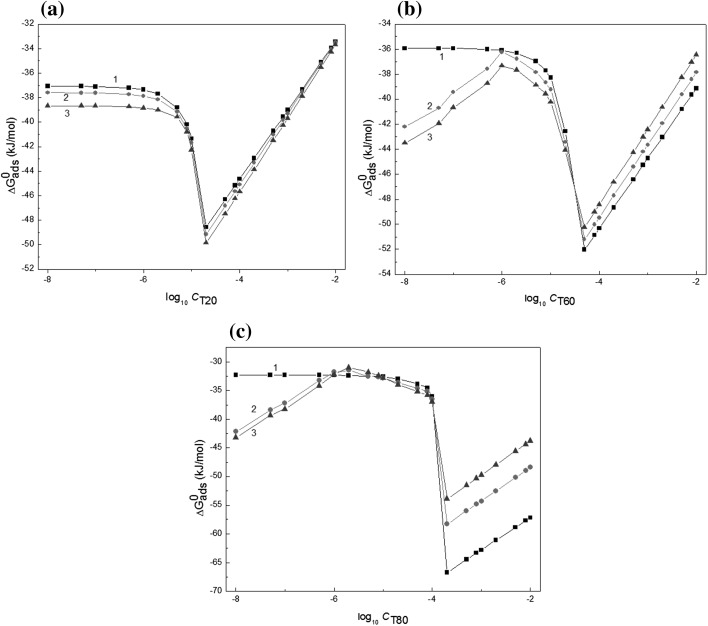
A plot of the standard Gibbs energy of adsorption ($\Delta G_{\text{ads}}^{\text{o}}$) of T20 (**a**), T60 (**b**) and T80 (**c**) at the water–air interface calculated from Eq. 10 against the logarithm of their concentration (log_10_ *C*). Curves 1, 2 and 3 correspond to the temperature equal to 293, 303 and 313 K, respectively

The values of $\Delta G_{\text{ads}}^{\text{o}}$ calculated from Eq. 10 are presented in Fig. 6 and those calculated from Eqs. 11 and 13 in Table 2. From Table 2 it can be seen that the tendency to adsorb is somewhat different for each surfactant and depends on *T*. Comparing the values of $\Delta G_{\text{ads}}^{\text{o}}$ of Tweens to those of classical nonionic surfactants such as Tritons [24], it can be stated that the tendency of Tweens to adsorb at the water–air interface is somewhat lower than that of the Tritons.

**Table 2:** The values of standard Gibbs energy of Tweens adsorption at the water–air interface ($\Delta G_{\text{ads}}^{\text{o}}$) calculated from Eqs. 10, 11, 13 and 15, as well as the standard enthalpy ($\Delta H_{\text{ads}}^{\text{o}}$) and the standard entropy of adsorption ($\Delta S_{\text{ads}}^{\text{o}}$) calculated from Eqs. 7 and 8, respectively

	*T* = 293 K	*T* = 303 K	*T* = 313 K
T20			
$\Delta G_{\text{ads}}^{\text{o}}$ (Eq. 10) [kJ·mol^−1^]	− 37.25 ± 0.43	− 37.77 ± 0.38	− 38.76 ± 0.34
$\Delta G_{\text{ads}}^{\text{o}}$ (Eq. 11) [kJ·mol^−1^]	− 39.68 ± 0.52	− 40.36 ± 0.48	− 41.40 ± 0.53
$\Delta G_{\text{ads}}^{\text{o}}$ (Eq. 13) [kJ·mol^−1^]	− 39.61 ± 0.55	− 40.91 ± 0.53	− 42.05 ± 0.51
$\Delta G_{\text{ads}}^{\text{o}}$ (Eq. 15) [kJ·mol^−1^]	− 38.23	–	–
$\Delta H_{\text{ads}}^{\text{o}}$ (Eq. 7) [kJ·mol^−1^]	− 3.864	− 3.944	− 3.864
$\Delta S_{\text{ads}}^{\text{o}}$ (Eq. 8) [kJ·mol·K^−1^]	0.122		
T60			
$\Delta G_{\text{ads}}^{\text{o}}$ (Eq. 10) [kJ·mol^−1^]	− 36.04 ± 0.21	− 38.83 ± 0.63	− 39.90 ± 0.71
$\Delta G_{\text{ads}}^{\text{o}}$ (Eq. 11) [kJ·mol^−1^]	− 38.06 ± 0.63	− 39.85 ± 0.68	− 41.09 ± 0.70
$\Delta G_{\text{ads}}^{\text{o}}$ (Eq. 13) [kJ·mol^−1^]	− 38.53 ± 0.73	− 39.54 ± 0.75	− 41.14 ± 0.76
$\Delta G_{\text{ads}}^{\text{o}}$ (Eq. 15) [kJ·mol^−1^]	− 37.64	–	–
$\Delta H_{\text{ads}}^{\text{o}}$ (Eq. 7) [kJ·mol^−1^]	− 1.64	− 4.29	− 4.29
$\Delta S_{\text{ads}}^{\text{o}}$ (Eq. 8) [kJ·mol·K^−1^]	0.114		
T80			
$\Delta G_{\text{ads}}^{\text{o}}$ (Eq. 10) [kJ·mol^−1^]	− 32.76 ± 0.20	− 34.66 ± 0.28	− 35.01 ± 0.31
$\Delta G_{\text{ads}}^{\text{o}}$ (Eq. 11) [kJ·mol^−1^]	− 33.28 ± 0.27	− 34.27 ± 0.32	− 34.50 ± 0.36
$\Delta G_{\text{ads}}^{\text{o}}$ (Eq. 13) [kJ·mol^−1^]	− 35.89 ± 0.41	− 37.34 ± 0.45	− 38.74 ± 0.47
$\Delta G_{\text{ads}}^{\text{o}}$ (Eq. 15) [kJ·mol^−1^]	− 37.64	–	–
$\Delta H_{\text{ads}}^{\text{o}}$ (Eq. 7) [kJ·mol^−1^]	0.35	− 0.42	− 0.36
$\Delta S_{\text{ads}}^{\text{o}}$(Eq. 8) [kJ·mol·K^−1^]	0.113		

It was proved earlier that the tendency of surfactants to adsorb at the water–air interface depends on their surface tension [30]. According to van Oss and Constanzo [20], the surface tension of surfactant can be divided into that of tail and head. The surface tension of the hydrocarbon tail results from the Lifshitz–van der Waals intermolecular interactions, and that of the hydrophilic head from the Lifshitz–van der Waals, Lewis acid–base and electrostatic interactions. In the case of Tweens, the electrostatic interactions can be neglected. Taking this into account it can be deduced that the standard Gibbs energy of adsorption fulfills the equation [30]:

14 $$\Delta G_{\text{ads}}^{\text{o}} = N\left[ {A_{\text{T}} \left( {\gamma_{\text{T}} - \gamma_{\text{WT}} } \right)} \right] + A_{\text{H}} \left( {\gamma_{\text{WH1}} - \gamma_{\text{WH}} } \right)$$

where $\gamma_{\text{T}}$ is the surface tension of surfactant tail and $\gamma_{\text{H}}$ is the surface tension of surfactant head, $\gamma_{\text{WT}}$ is the tail–water interface tension, $\gamma_{\text{WH}}$ and $\gamma_{\text{WH1}}$ are the water–head interface tensions for the hydrated and dehydrated forms of the head, $A_{\text{T}}$ is the contactable area of the surfactant tail or its part, and $A_{\text{H}}$ is the contactable area of the surfactant head or head with a part of the tail. If during the adsorption of surfactant its molecules are not dehydrated, then [30]:

15 $$\Delta G_{\text{ads}}^{\text{o}} = NA_{\text{T}} \left( {\gamma_{\text{T}} - \gamma_{\text{WT}} } \right)$$

From Eq. 15 it results that determination of $\Delta G_{\text{ads}}^{\text{o}}$ requires the values of the tail surface tension, tail–water interface tension and contactable area of tail. It was assumed earlier that the surface tension of the tail corresponds to that of hydrocarbon as tail [30] and the tail–water interface tension corresponds to that of a hydrocarbon–water interface. The contactable area of the tail can be deduced from the bonds length and angles between them as well as the average distance between the molecules being in contact [21]. In the case of T20 it was assumed that its tail corresponds to *n*-undecane, for T60 to *n*-heptadecane and for T80 for heptadecene. The surface tension of *n*-undecane is equal to 24.7 mN·m^−1^ and *n*-undecane–water interface tension is equal to 51.1 mN·m^−1^ [31]. One can find in the literature that the surface tensions of *n*-heptadecane and heptadecene have the same value, equal to 26.9 mN·m^−1^ [32]. However, it is difficult to find the values of *n*-heptadecane–water and heptadecene–water interface tension. At the first approximation the $\gamma_{\text{WT}}$ values can be predicted on the basis of the Young equation which has the form [28]:

16 $$\gamma_{\text{S}} - \gamma_{\text{S/L}} = \gamma_{\text{L}} \cos \theta$$

where $\gamma_{\text{S}}$ is the solid surface tension, $\gamma_{\text{S/L}}$ is the solid–liquid interface tension and $\theta$ is the contact angle.According to van Oss et al. [33–36] the Young equation for a hydrophobic solid can be written as:

17 $$\gamma_{\text{L}} \cos \theta = - \gamma_{\text{L}} + 2\sqrt {\gamma_{\text{L}}^{\text{LW}} \gamma_{\text{S}}^{\text{LW}} }$$

where $\gamma_{\text{L}}^{\text{LW}}$ and $\gamma_{\text{S}}^{\text{LW}}$ are the Lifshitz–van der Waals component of the liquid and solid surface tensions, respectively.

Introducing into Eq. 17 the surface tension of water is equal to 72.8 mN·m^−1^ at 293 K, the Lifshitz–van der Waals component of its tension is equal to 26.85 mN·m^−1^ [37] as well as the surface tension of heptadecane that is equal to 26.9 mN·m^−1^ instead of $\gamma_{\text{S}}^{\text{LW}},$ then the contact angle of water was calculated to be 105.2°. Next, $\gamma_{\text{S/L}}^{\text{LW}}$ was determined to be 45.95 mN·m^−1^. On the basis of these data it was possible to calculate the $\Delta G_{\text{ads}}^{\text{o}}$ for Tweens from Eq. 15. The calculations were made on the assumption that the tails of the surfactant are oriented parallel to the water–air interface, which is possible in the unsaturated monolayer. In such a case one side of a tail is in contact with water. The values of $\Delta G_{\text{ads}}^{\text{o}}$ calculated from Eq. 15 are presented in Table 2. As follows from this table, the values of $\Delta G_{\text{ads}}^{\text{o}}$ calculated in such way are comparable to those determined from the Langmuir and Rosen equations.

Knowing the values of $\Delta G_{\text{ads}}^{\text{o}}$ at different temperatures it was possible to determine $\Delta H_{\text{ads}}^{\text{o}}$ and $\Delta S_{\text{ads}}^{\text{o}}$ (Eqs. 7–9). As can be seen from Table 2 the values of $\Delta H_{\text{ads}}^{\text{o}}$ increase from T20 to T80. For T80 the $\Delta H_{\text{ads}}^{\text{o}}$ values are close to zero. The $\Delta H_{\text{ads}}^{\text{o}}$ values for all the studied Tweens indicate that there is probably compensation of the effects of surfactants tail and head dehydration and that these effects are comparable. This fact confirms the conformability between the calculated values of $\Delta G_{\text{ads}}^{\text{o}}$ from Eq. 15 and those calculated from the Langmuir and Rosen equations [18, 28].

### Thermodynamic Functions of Tweens Micellization

The values of the CMC determined from the surface tension isotherms of aqueous Tween solutions (Figs. 2, 3, and 4, Table 3) decrease as a function of temperature as well as from T20 to T80. However, the CMC values determined by us are higher than those reported in the literature [8–17]. Comparing the CMC values obtained from the isotherm of surface tension to those determined by other methods, it can be stated that there is no agreement between these values [8–17, 19]. Unfortunately, the values of CMC obtained here from the isotherm of surface tension, which was measured by the weight–volume method, are considerably higher than those obtained from the isotherm of surface tension measured by the ring method [8, 10, 11]. The CMC values of Tweens are comparable to those of other surfactants including oxyethylene groups in the hydrophilic part of molecules [38]. The tendency of Tweens to form the micelles and the energetic changes in the system occurred during the micellization process can be deduced on the basis of the standard Gibbs energy ($\Delta G_{\text{mic}}^{\text{o}}$), enthalpy ($\Delta H_{\text{mic}}^{\text{o}}$) and entropy ($\Delta S_{\text{mic}}^{\text{o}}$) of micellization. For determination of $\Delta G_{\text{mic}}^{\text{o}}$ for nonionic surfactants, the following equation is very often used [18]:

18 $$\Delta G_{\text{mic}}^{\text{o}} = RT\ln \left( {\frac{{C_{\text{CMC}} }}{\omega }} \right)$$

**Table 3:** The values of critical micelle concentration of Tweens (CMC) and Gibbs standard energy of micellization ($\Delta G_{\text{mic}}^{\text{o}}$) calculated from Eqs. 18 and 21 as well as the standard enthalpy ($\Delta H_{\text{mic}}^{\text{o}}$) and the standard entropy of micellization ($\Delta S_{\text{mic}}^{\text{o}}$) calculated based on Eqs. 7 and 8, respectively

	Temperature [K]		
	293	303	313
T20			
CMC [mol·dm^−3^]	9.75 × 10^−4^	9.42 × 10^−4^	9.18 × 10^−4^
$\Delta G_{\text{mic}}^{\text{o}}$ (Eq. 18) [kJ·mol^−1^]	− 26.67	− 27.66	− 28.63
$\Delta G_{\text{mic}}^{\text{o}}$ (Eq. 21) [kJ·mol^−1^]	− 28.17	–	–
$\Delta H_{\text{mic}}^{\text{o}}$ (Eq. 7) [kJ·mol^−1^]	2.04	2.03	2.04
$\Delta S_{\text{mic}}^{\text{o}}$ (Eq. 8) [kJ·mol^−1^·K^−1^]	0.098		
T60			
CMC [mol·dm^−3^]	7.48 × 10^−4^	8.25 × 10^−4^	7.29 × 10^−^
$\Delta G_{\text{mic}}^{\text{o}}$ (Eq. 18) [kJ·mol^−1^]	− 27.32	− 27.99	− 29.23
$\Delta G_{\text{mic}}^{\text{o}}$ (Eq. 21) [kJ·mol^−1^]	− 28.38	–	–
$\Delta H_{\text{mic}}^{\text{o}}$ (Eq. 7) [kJ·mol^−1^]	0.52	0.80	0.51
$\Delta S_{\text{mic}}^{\text{o}}$ (Eq. 8) [kJ·mol^−1^·K^−1^]	0.095		
T80			
CMC [mol·dm^−3^]	5.74 × 10^−4^	4.41 × 10^−4^	4.39 × 10^−4^
$\Delta G_{\text{mic}}^{\text{o}}$ (Eq. 18) [kJ·mol^−1^]	− 27.96	− 29.57	− 30.55
$\Delta G_{\text{mic}}^{\text{o}}$ (Eq. 21) [kJ·mol^−1^]	− 32.52	–	–
$\Delta H_{\text{mic}}^{\text{o}}$ (Eq. 7) [kJ·mol^−1^]	0.75	0.12	0.12
$\Delta S_{\text{mic}}^{\text{o}}$ (Eq. 8) [kJ·mol^−1^·K^−1^]	0.098		

It should be remembered that this equation was drawn on the assumption that at the concentration of surfactants in the bulk phase equal to CMC, the activity coefficient ($f$) is close to unity when the chemical potential of surfactants is asymmetrically defined. This means that if $\frac{{C_{\text{CMC}} }}{\omega } \approx X_{\text{CMC}} \to 0$, then $f \to 1$. The calculated values of $\Delta G_{\text{mic}}^{\text{o}}$ (Table 3) depend on the type of Tween and temperature. These values are somewhat higher than those for Tritons [38]. Knowing the values of $\Delta G_{\text{mic}}^{\text{o}}$ it is possible to determine $\Delta H_{\text{mic}}^{\text{o}}$ and $\Delta S_{\text{mic}}^{\text{o}}$ on the assumption that in the range of studied temperatures $\Delta H_{\text{mic}}^{\text{o}}$ or $\Delta S_{\text{mic}}^{\text{o}}$ is constant (the same Eqs. as Eqs. 7–9). The calculated values of $\Delta H_{\text{mic}}^{\text{o}}$ and $\Delta S_{\text{mic}}^{\text{o}}$ are presented in Table 3. From this Table it is clear that that there is a small positive enthalpy, which is close to zero from T20 to T80. The values of the enthalpy close to zero can suggest that the positive values of enthalpy of dehydration of surfactant molecules head is compensated by a negative value connected with the dehydration of surfactant molecules tail. During the hydration of surfactant heads, hydrogen bonds are broken. This can result in a positive enthalpy change. On the other hand, the water molecules around the surfactant tail, after their adsorption at the water–air interface, form the water structure by hydrogen bonding, releasing energy (negative enthalpy change).

The standard Gibbs energy of micellization should be combined with the Gibbs energy of interactions of Tweens molecules through the water phase [38]. According to the extended DLVO theory [20], the total Gibbs energy of interactions through the water between the two identical particles or moieties, $\Delta G_{{1{\text{W}}1}}^{\text{Tot}}$, is:

19 $$\Delta G_{{ 1 {\text{W1}}}}^{\text{Tot}} = \Delta G_{{ 1 {\text{W1}}}}^{\text{LW}} + \Delta G_{{ 1 {\text{W1}}}}^{\text{AB}} + \Delta G_{{ 1 {\text{W1}}}}^{\text{EL}}$$

where LW, AB and EL refer to the contributions to the Gibbs energy coming from the interactions resulting from the Lifshitz–van der Waals, Lewis acid–base and electrostatic forces, and the subscripts 1 and W refer to the particles or moieties and water, respectively. For Tweens which are nonionic surfactants there can be written [38]:

20 $$\Delta G_{{ 1 {\text{W1}}}}^{\text{Tot}} = \Delta G_{{ 1 {\text{W1}}}}^{\text{LW}} + \Delta G_{{ 1 {\text{W1}}}}^{\text{AB}}$$

or

21 $$\Delta G_{{ 1 {\text{W1}}}}^{\text{Tot}} = - 2\left( {\gamma_{\text{WT}} + \gamma_{\text{WH}} } \right)$$

Knowing the components and parameters of water and head-group surface tension [21] it is possible to calculate $\gamma_{\text{WH}}$ from the following equation [33–36]:

22 $$\gamma_{\text{WH}} = \gamma_{\text{W}} + \gamma_{\text{H}} - 2\left( {\sqrt {\gamma_{\text{W}}^{\text{LW}} \gamma_{\text{H}}^{\text{LW}} } + \sqrt {\gamma_{\text{W}}^{ + } \gamma_{\text{H}}^{ - } } + \sqrt {\gamma_{\text{W}}^{ - } \gamma_{\text{H}}^{ + } } } \right)$$

Knowing the size of the contactable area of tails, *S*_T_ and heads, *S*_H_ of the surfactant molecule, it is possible to calculate Gibbs energy of interactions between two molecules of surfactant through the water and, taking into account the Avogadro number, it is possible to calculate the Gibbs energy of interaction in water corresponding to one mole of surfactant from the following equation:

23 $$\Delta G_{\text{inter}}^{\text{o}} = - 2N\left( {\gamma_{\text{WT}} S_{\text{T}} + 2\gamma_{\text{WH}} S_{\text{T}} } \right)$$

This energy should correspond to the standard Gibbs energy of micellization. It appears that the values of $\Delta G_{\text{inter}}^{\text{o}}$ calculated for Tweens at *T* = 293 K are close to those of the standard Gibbs energy of micellization.

## Conclusions

On the basis of the obtained results and their discussion it can be stated that:

(i) The packing of the Tween molecules in the monolayer at the water–air interface is larger than of other nonionic surfactants. This means that there are practically no repulsive interactions between Tween molecules in the saturated monolayer.
(ii) The packing of the Tween molecules in the saturated monolayer increases from T20 to T80 and depends on the conformation of the head of the molecules.
(iii) The packing of the molecules of each Tween depends on the temperature.
(iv) The standard Gibbs energy of Tween adsorption depends on the kind of Tween and temperature. An increase of temperature causes a decrease of the Gibbs energy of adsorption.
(v) The standard Gibbs energy of adsorption can be predicted from the surface tension of tail of Tween molecules and the water–tail interface tension.
(vi) The standard enthalpy of Tween adsorption is negative and increases from T20 to T80. The values of enthalpy suggest that the process of dehydration of tail and head gives compensating effects because the absolute values of negative and positive enthalpy are nearly the same.
(vii) The CMC values of the Tweens determined from the surface tension isotherms are comparable to those of the classical nonionic surfactants but they are higher than those reported in the literature.
(viii) The CMC values depend on the type of Tween and temperature. The CMC decreases slightly with increasing temperature.
(ix) The thermodynamic functions of Tween micellization indicate that this process results from dehydration of tails and heads.
(x) The standard Gibbs energy of micellization can be predicted based on the tail and head surface tension.

## Electronic supplementary material

Below is the link to the electronic supplementary material.

Supplementary material 1 (DOC 92 kb)
