# Supplementary material for: Adsorption and Aggregation Properties of Some Polysorbates at Different Temperatures
Source: J Solution Chem. 2018 Oct 26;47(11):1824–40. doi: 10.1007/s10953-018-0823-z (PMC6244871; doi:10.1007/s10953-018-0823-z)
Supplement: Supplementary file 1 — Supplementary material 1 (DOC 92 kb) [file 10953_2018_823_MOESM1_ESM.doc]

**ADSORPTION AND AGGREGATION PROPERTIES OF SOME POLYSORBATES AT DIFFERENT TEMPERATURES**

KATARZYNA SZYMCZYK*. ANNA ZDZIENNICKA AND BRONISŁAW JAŃCZUK

*Department of Interfacial Phenomena. Faculty of Chemistry. Maria Curie-Skłodowska University. Maria Curie-Skłodowska Sq. 3. 20-031 Lublin. Poland*

Running title: Adsorption and aggregation properties

*To whom correspondence should be addressed

phone (48-81) 537-56-70

fax (48-81) 533-3348

e-mail [katarzyna.szymczyk@poczta.umcs.lublin.pl](mailto:katarzyna.szymczyk@poczta.umcs.lublin.pl)

**Table 1** The values of the surface tension (
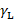
) of the aqueous solution of Tween 20 (T20). Tween 60 (T60) and Tween 80 (T80) at 293, 303 and 313 K.

| log_10_*C* | 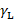 [mN·m^−1^] | | | | | | | | |
| --- | --- | --- | --- | --- | --- | --- | --- | --- | --- |
|  | T20 | | | T60 | | | T80 | | |
|  | *T* [K] | | | | | | | | |
|  | 293 | 303 | 313 | 293 | 303 | 313 | 293 | 303 | 313 |
| −8.0 | 71.86 | 70.35 | 68.83 | 71.86 | 70.35 | 68.83 | 71.30 | 69.79 | 68.27 |
| −7.3 | 71.83 | 70.32 | 68.80 | 71.78 | 70.27 | 68.75 | 70.73 | 69.22 | 67.70 |
| −7.0 | 71.80 | 70.29 | 68.77 | 71.70 | 70.19 | 68.67 | 70.55 | 69.04 | 67.52 |
| −6.3 | 71.61 | 70.10 | 68.58 | 71.48 | 69.97 | 68.45 | 69.94 | 68.43 | 66.91 |
| −6.0 | 71.38 | 69.87 | 68.35 | 71.28 | 69.77 | 68.25 | 69.67 | 68.16 | 66.64 |
| −5.7 | 70.91 | 69.40 | 67.88 | 71.13 | 69.62 | 68.10 | 69.43 | 67.92 | 66.40 |
| −5.3 | 69.39 | 67.88 | 66.36 | 69.30 | 67.79 | 66.27 | 69.12 | 67.61 | 66.09 |
| −5.1 | 67.53 | 66.02 | 64.50 | 68.70 | 67.19 | 65.67 | 68.78 | 67.27 | 65.75 |
| −5.0 | 66.10 | 64.59 | 63.07 | 67.40 | 65.89 | 64.37 | 68.6 | 67.09 | 65.57 |
| −4.7 | 61.74 | 60.23 | 58.71 | 64.10 | 62.59 | 61.07 | 67.02 | 65.51 | 63.99 |
| −4.3 | 55.70 | 54.19 | 52.67 | 58.00 | 56.49 | 54.97 | 61.22 | 59.71 | 58.19 |
| −4.1 | 51.92 | 50.41 | 48.89 | 54.30 | 52.79 | 51.27 | 57.12 | 55.61 | 54.09 |
| −4.0 | 50.63 | 49.12 | 47.60 | 52.90 | 51.39 | 49.87 | 54.83 | 53.32 | 51.80 |
| −3.7 | 45.81 | 44.30 | 42.78 | 47.76 | 46.25 | 44.73 | 48.00 | 46.49 | 44.97 |
| −3.3 | 39.41 | 37.90 | 36.38 | 41.00 | 39.49 | 37.97 | 42.19 | 40.68 | 39.16 |
| −3.1 | 36.92 | 35.41 | 33.89 | 38.90 | 37.39 | 35.87 | 40.28 | 38.77 | 37.25 |
| −3.0 | 36.27 | 34.76 | 33.24 | 38.86 | 37.35 | 35.83 | 39.95 | 38.44 | 36.92 |
| −2.7 | 35.15 | 33.64 | 32.12 | 38.00 | 36.49 | 34.97 | 39.71 | 38.20 | 36.68 |
| −2.3 | 35.00 | 33.49 | 31.97 | 37.90 | 36.39 | 34.87 | 39.60 | 38.09 | 36.57 |
| −2.1 | 34.92 | 33.41 | 31.89 | 37.85 | 36.34 | 34.82 | 39.55 | 38.04 | 36.52 |
| −2.0 | 34.90 | 33.39 | 31.87 | 37.85 | 36.34 | 34.82 | 39.50 | 37.99 | 36.47 |

The standard deviation of
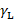
 is changed as a function of *C* from ± 0.1 to ± 0.25 mN·m^−1^
